# The growth pattern of the human intestine and its mesentery

**DOI:** 10.1186/s12861-015-0081-x

**Published:** 2015-08-22

**Authors:** Jelly HM Soffers, Jill PJM Hikspoors, Hayelom K. Mekonen, S. Eleonore Koehler, Wouter H. Lamers

**Affiliations:** Department of Anatomy & Embryology, Maastricht University, P.O. Box 616, 6200 MD Maastricht, The Netherlands; Tytgat Institute for Liver and Intestinal Research, Academic Medical Center, University of Amsterdam, Amsterdam, The Netherlands

## Abstract

**Background:**

It remains unclear to what extent midgut rotation determines human intestinal topography and pathology. We reinvestigated the midgut during its looping and herniation phases of development, using novel 3D visualization techniques.

**Results:**

We distinguished 3 generations of midgut loops. The topography of primary and secondary loops was constant, but that of tertiary loops not. The orientation of the primary loop changed from sagittal to transverse due to the descent of ventral structures in a body with a still helical body axis. The 1^st^ secondary loop (duodenum, proximal jejunum) developed intraabdominally towards a left-sided position. The 2^nd^ secondary loop (distal jejunum) assumed a left-sided position inside the hernia before returning, while the 3^rd^ and 4^th^ secondary loops retained near-midline positions. Intestinal return into the abdomen resembled a backward sliding movement. Only after return, the 4^th^ secondary loop (distal ileum, cecum) rapidly “slid” into the right lower abdomen. The seemingly random position of the tertiary small-intestinal loops may have a biomechanical origin.

**Conclusions:**

The interpretation of “intestinal rotation” as a mechanistic rather than a descriptive concept underlies much of the confusion accompanying the physiological herniation. We argue, instead, that the concept of “en-bloc rotation” of the developing midgut is a fallacy of schematic drawings. Primary, secondary and tertiary loops arise in a hierarchical fashion. The predictable position and growth of secondary loops is pre-patterned and determines adult intestinal topography. We hypothesize based on published accounts that malrotations result from stunted development of secondary loops.

**Electronic supplementary material:**

The online version of this article (doi:10.1186/s12861-015-0081-x) contains supplementary material, which is available to authorized users.

## Background

Congenital intestinal malrotations are thought to be present in 1:200–500 live births [1]. Malrotations are considered as incomplete or abnormal rotations of the midgut around the vitelline/superior mesenteric artery (SMA). The symptoms of malrotation are not well understood and not all malrotations become symptomatic [2, 3]. If malrotations become symptomatic, it is usually in the early months of life, with 75–85 % of cases diagnosed within one year of age [4]. If one assumes that malrotations represent “frozen” stages of normal development [5], it becomes important to establish how the intestine develops.

The standard description of normal intestinal “rotation” encompasses a couple of sequential developmental steps. Initially, the midgut loop forms and extends into the extra-abdominal cavity due to rapid growth of this part of the intestine. This process is known as physiological intestinal herniation. Further development of this loop is associated with a change in position of the small intestine from cranial to right-sided, and of the cecum from caudal to left-sided, that is, with an apparent ~90° counterclockwise rotation. The duodenojejunal junction acquires its left-sided position during the herniation phase of development, whereas the cecum acquires its right-sided position upon return of the gut into the abdominal cavity. Together, these changes in position would represent an additional ~180° counterclockwise rotation. However, few of these largely schematic descriptions and illustrations are based on original studies (e.g., [6–8]. Moreover, the authors of one of the first studies already cautioned that “it must not been assumed that the main vessel, the SMA, actually forms an axis around which the loop twists” [6]. This so-called rope model with an “en-bloc rotation” of the gut over 270° around the SMA has, nevertheless, become the current paradigm in virtually all texts. Despite a century of studies, it remains unclear to what extent active rotation, or its absence, contributes to intestinal development [9].

Another hallmark of intestinal development is the formation of the intestinal coils. Although the shape and position of the early loops appear very constant [10–13], it is widely assumed that the number and position of the small intestinal coils are random. An invariable pattern of loop formation during the early herniation period implies the presence of a pre-pattern, whereas a random pattern is compatible with a stochastic control of loop formation. The tensile interaction between the intestinal tube and its suspending mesentery was recently proposed as a potential mechanism for stochastic coil formation [14].

If we are to resolve these apparent contradictions, it becomes important to establish what part of intestinal looping is hard-wired in a genetically determined pre-pattern and what part is not. We, therefore, reinvestigated the looping pattern of the human intestine and provide virtual 3D reconstructions of the intestinal loops, their blood supply and mesenteric suspension in human embryos during and after herniation into the hernial sac. The results support the existence of a pre-pattern for the establishment of the intestinal loops during the early herniation period, which in turn largely determines the topography of the intestine in the abdominal cavity after the umbilical herniation has resolved. In contrast, the loops that are formed during and after the late herniation period appear to form under stochastic control.

## Results

### The pre-herniation period

**Fig. 1:**
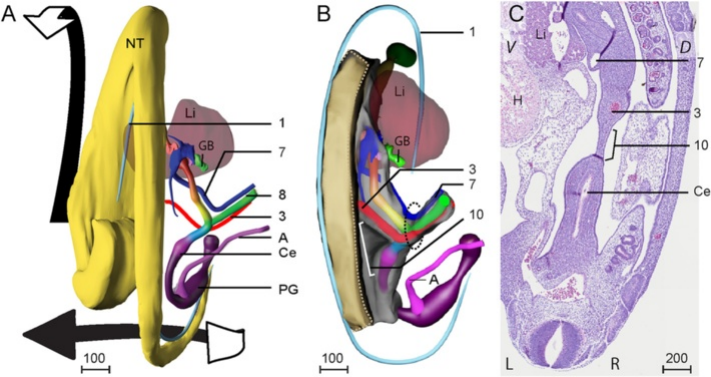
The orientation of the midgut loop and its mesentery during the 5^th^ week follows the helical body axis. Panel **a**: Dorsal view of the reconstruction of a CS14 embryo (s5029). Note the left-sided juxtaposition of the head relative to the caudal end of the body, reflecting the helical body axis. The successive parts of the midgut are shown in a rainbow color gradient (see legend color codes). Note that the vitelline artery (3) and the right vitelline vein (7) traverse the vitelline duct (8) at the apex of the midgut loop. The arrows indicate the changes occurring during straightening of the body axis in CS15 and CS16 embryos. Panel **b** shows the position of the developing midgut mesentery (10) between both limbs of the midgut. Note the limited craniocaudal extension of the mesentery at this stage (10). The beige area identifies the region where the intestinal mesenchyme is attached to the dorsal body wall. Panel **c**: Histological section of embryo s5029 with right vitelline vein (7), vitelline artery (3), cecum (Ce) and developing dorsal midgut mesentery (10). Due to the helical body axis, the caudal end of the body is cut near transversely (with left (L) and right (R) sides), whereas more cranially, the body is cut almost sagittally (V: ventral; D: dorsal). Note that the midgut mesentery (10) is ~4-fold thinner than the mesenchymal mass surrounding the intestine. Scale bar unit: μm. An interactive 3D-PDF is available online (3D-PDF CS14)

The foregut could first be identified in Carnegie Stage (CS) 9 embryos (25–27 days post fertilization) and the hindgut at CS10 (28–30 days). The rapid longitudinal growth of the embryo in the 5^th^ developmental week, reflected in a 5-fold increase in greatest length [15], resulted in a corkscrew-like appearance of the longitudinal body axis. The caudal portion of the embryo was always located to the right of the head (Fig. 1a). Since both fore- and hindgut were midline structures in this period, their longitudinal axis followed that of the body (see 3D-PDF CS14). The initial connection of the intestine with the yolk sac was wide and located at the right side. This connection grew less rapidly than the embryo proper and became the tubular vitelline duct (Fig. 1a; [8]).

#### The formation of the midgut (week 5)

**Fig. 2:**
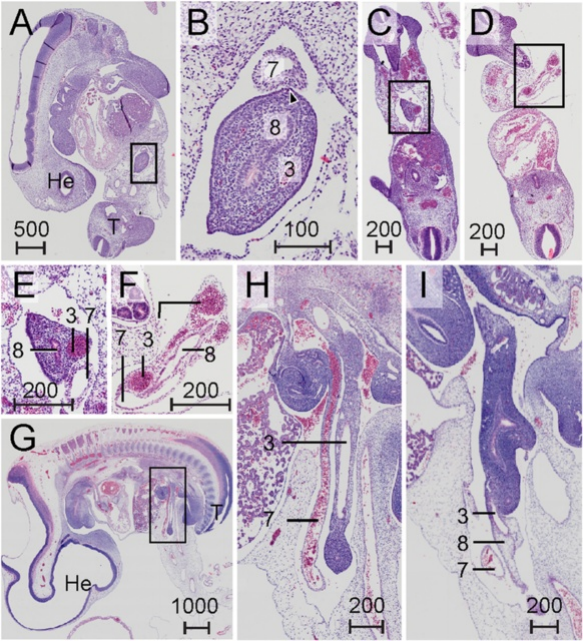
Landmark structures for the apex of the midgut loop. Histological section of a CS14 embryo (s5029, panel **a**) and magnification of the boxed region (panel **b**) showing the very thin connection (arrowhead) between the right vitelline vein (7) and the midgut loop near its apex. Panels **c** and **d**: histological sections of a CS14 embryo (s2201). Panel **e**: Magnification of the boxed region from C to show the proximity of the right vitelline vein (7) and the vitelline duct (8). Panel **f**: Magnification of the boxed region from D to show that inside the umbilicus the open vitelline duct (8), vitelline artery (3) and right vitelline vein (7) are surrounded by the periductal mesenchyme. Panel **g**: Midsagittal histological section of a CS16 embryo (s5032). Panel **h**: The magnification of the boxed area from panel **g**. The right vitelline vein (7) lost its connection with the midgut and courses as a free vessel in the body cavity. Panel **i**: The magnification of the same region in G from a more lateral section to show that the vitelline duct is no longer present as open duct inside the periductal mesenchyme (81). Scale bar units: μm

The intestine started to loop ventrally during CS14 (33–35 days), with the connection with the vitelline duct at its apex (Fig. 1a, Fig. 2a,b). The connection of this “primary” loop with the dorsal body wall was visibly thinner than the comparable connections of more cranial or caudal parts of the gut, and was, therefore, already identifiable as the dorsal mesentery of the midgut (Fig. 1b,c). The cranial end of the midgut loop (color coded orange) was located between segments 11 and 13 (corresponding to Th4-6; for numbering of segments, see Methods) between the confluence of both vitelline veins and the origin of the vitelline artery from the aorta (Fig. 1a; see also 3D-PDF CS14). The caudal end of the midgut loop was found caudal to a local widening of the midgut, the future cecum, at the level of segments 17–18 (Th10-11; Fig. 1a,c). The plane through the midgut loop and its mesentery remained midsagittal, that is, followed that of the helical body axis (see 3D-PDF CS14).

#### Landmarks for the apex of the midgut loop

The midgut is that part of the intestine which forms the primary loop. Later in development, when vessels can be distinguished, it coincides with the distribution area of the superior mesenteric artery (SMA, see the section on “The branching pattern of the superior mesenteric artery”). The vitelline artery and duct, unambiguous landmarks for the apex of the midgut, were located inside the “periductal” mesenchyme that surrounded the connection of the intestine with the yolk sac (Fig. 1b,c). The vitelline duct became interrupted at CS15 (35–37 days) and the vitelline artery no longer extended as a patent vessel into the periductal mesenchyme at CS16 (37–40 days). We, therefore, designate its remaining proximal part as the SMA from CS16 onwards. Another conspicuous vessel in the periductal mesenchyme that is rarely described in literature was the right vitelline vein, which drained blood from the yolk sac. The right vitelline vein passed the midgut apex and proximal midgut limb cranially. At CS14, the vessel was attached to, but not embedded in the connective tissue surrounding the intestinal tube (Fig. 2a,b), but in subsequent stages, the vein became disconnected from the intestinal mesenchyme, so that it appeared as a “free” vessel in the abdominal cavity (Fig. 2d-f). The left vitelline vein drained the midgut and was found inside the mesentery between the proximal and distal limbs of the primary loop. Just caudal to the pancreas, both veins merged. The periductal mesenchyme and the right vitelline vein continued to mark the apex of the midgut loop until shortly after the return of the herniated intestine into the abdominal cavity.

### The herniation period

#### The herniation of the primary midgut loop (week 6)

**Fig. 3:**
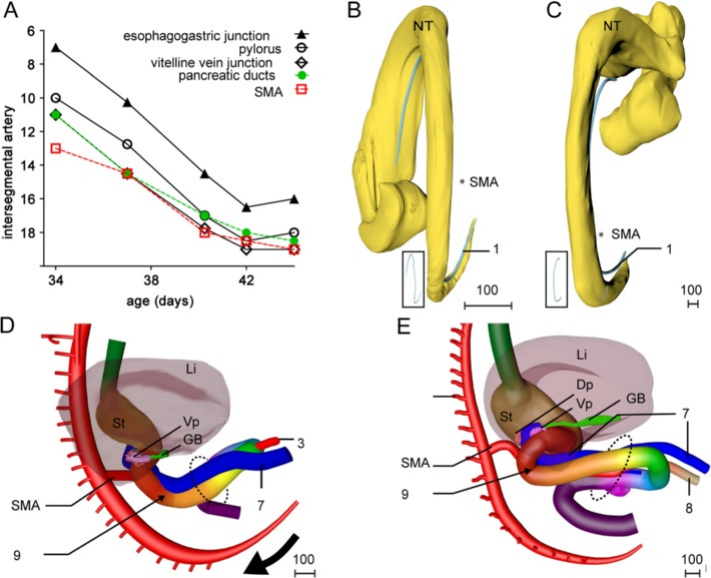
Descent of the ventral organs during the 5^th^ and 6^th^ week moves the proximal limb of the primary midgut loop rightward. Panel **a**: Descent of structures in the upper abdomen between CS14 and CS18 relative to the intersegmental arteries. Intersegmental artery 7 identifies vertebra C7. Panels **b** and **c**: Dorsal views of reconstructions of a CS14 (s5029) and a CS16 (s5032) embryo, respectively, with the notochord (1) and neural tube (NT) aligned in their medial portions. Inset: notochord alone. The helical alignment of the body axis has largely resolved at CS16. Panels **d** and **e**: Right-sided views of reconstructions of the intestine at CS15 (2213) and CS16 (s5032), respectively. The cranial end of the dorsal midgut mesentery is identified by number 9. The right vitelline vein (7), the vitelline/SMA (3), and the periductal mesenchyme (8) mark the apical portion of the midgut. The umbilical orifice is indicated by a dashed oval. The plane through the orifice changed with the straightening of the body (*bold arrow* in panel **d**). Note that the descent of the ventral organs moves the proximal limb of the midgut loop to a more rightward and dorsal position. The orange numbers identify intersegmental artery #15 (corresponding to vertebra Th8). Scale bar unit: μm. An interactive 3D-PDF is available online (3D-PDF CS16)

Starting in the 5^th^ week and continuing during the 6^th^ week, the root of the SMA descended from the level of segment 13 to that of segment 19 (corresponding to Th6 and Th12, respectively). The position of the esophagogastric junction, pylorus, pancreatic ducts and confluence of both vitelline veins descended concomitantly, but over 8–9 segments (Fig. 3a). The descent was initiated when the body axis is still helical (Fig. 3b), so that a cranial midline structure that shifted caudally, also shifted to the right of the midline (Fig. 3c). As a result of the descent, the cranial limb of the midgut acquired a position to the right of and at same transverse level as the caudal limb (Fig. 3d,e). During the 6^th^ week, the helical appearance of the embryonic body axis resolved, concomitant with the straightening of the trunk (Fig. 3b,c). From this stage onwards, the umbilicus and the associated structures exited the embryo at its ventral side. The midgut loop increased further in length, so that its apex herniated into the umbilical coelom (Fig. 3d,e). The proximal midgut segment (red) did not develop a thin mesentery. The dorsal mesentery of the remaining part of the midgut transformed into a rod-shaped mesenchymal mass. At its ventral tip the rod remained connected with the periductal mesenchyme. The right vitelline vein and mesenteric rod formed the central axis of the primary loop (Figs. 2e-h, 3d,e and 3D-PDF CS16).

#### The appearance of four secondary loops (week 7)

**Fig. 4:**
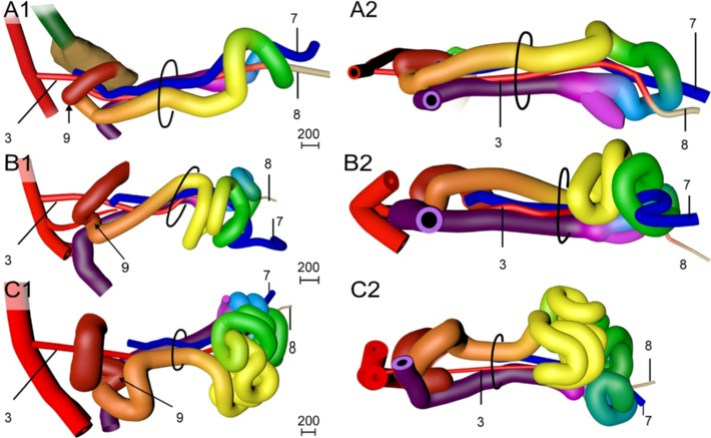
Development of secondary loops in the small intestine during the 7^th^ and 8^th^ week is invariant. *Right*-*lateral* (panels **a1**-**c1**) and caudal (panels **a2**-**c2**) views of the reconstructed intestines of CS18 (s97; panel **a**), CS20 (462; panel **b**), and CS23 (s4141; panel **c**) embryos. The beige strand (8) represents the periductal mesenchyme. Ovals: umbilical orifice. Note the dorsal (**a**) and then *leftward growth* (**b**, **c**) of the apex of the 1^st^ secondary loop (coded red and orange), followed by the formation of tertiary loops in its distal limb (**c**; *coded orange*). Further note the caudal (**a**) and then leftward growth (**b**, **c**) of the apex of the 2^nd^ and 3^rd^ secondary loops (coded *yellow and green*, respectively), followed by the formation of tertiary loops (**c**). Also note that the apex of the 4^th^ secondary loop (*coded blue*) grows cranially (**a**) before forming tertiary loops (**c**). Scale bar units: μm. An interactive 3D-PDF is available online (3D-PDF CS20)

**Fig. 5:**
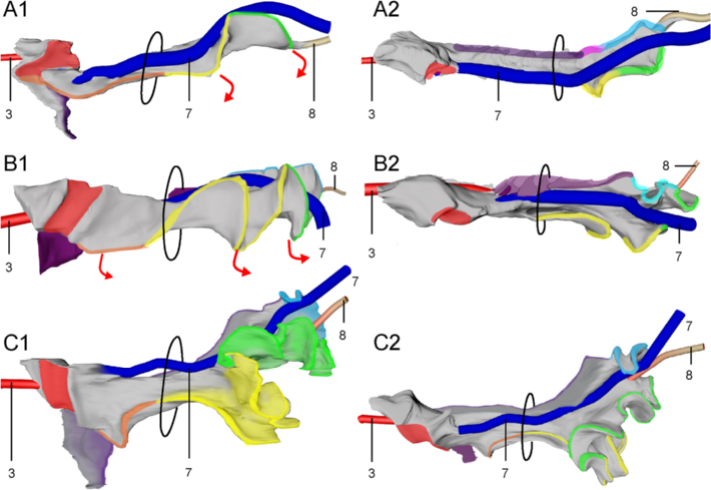
Mesenteric architecture accentuates boundaries of secondary loops. *Right*-*sided* (panels **a1**-**c1**) and cranial (panels **a2**-**c2**) views of the reconstructed midgut mesentery after removal of the intestinal tube at CS18 (s97; panel **a**), CS20 (462; panel **b**) and CS23 (s4141; panel **c**). The colors of the cut edges of the mesentery match the color code of the corresponding parts of the intestine. The mesocolon is shown in purple. Ovals: umbilical orifice. Note the central position of the right vitelline vein (7) along the central mesenteric rod. The formation of tertiary loops was associated with a lengthening of the mesenteric leaves (*red arrows* in panels **a1**, **b1**) that define the secondary loops (compare panels **b** and **c**). Interactive 3D-PDFs are available online (3D-PDFs CS20, CS23)

In the course of the late 6^th^ and 7^th^ weeks of development, the midgut increased ~1.4-fold in length and formed 4 secondary loops (Fig. 4). The 1^st^ secondary loop (coded red and orange) developed intra-abdominally at CS16 (Fig. 3d,e) between the duct of the ventral pancreas and the passage of the midgut through the umbilical orifice. Initially, this 1^st^ secondary loop expanded rightward and caudally on the right side of the SMA (Figs. 3d,e and 4). The other three secondary loops developed extra-abdominally during CS17 and CS18 (39–45 days; Figs. 3d,e and 4). The 2^nd^ and 3^rd^ loop (coded yellow and green, respectively) developed from the remaining part of the cranial limb of the midgut and expanded caudally and then leftward on the right side of the axis formed by the SMA, right vitelline vein, and mesenteric rod (Fig. 4). The distal end of this axis, i.e., the periductal mesenchyme and right vitelline vein, still marked the apex of the intestine. The 4^th^ loop (coded blue) developed from the caudal limb of the midgut between the apex and the cecum, and expanded cranially on the left side of the axis. Thin sideward extensions of the mesentery connected the intestinal tube with the mesenteric rod (Fig. 5a). The mesenteric leaf on the right side of the mesenteric rod connected with loops 2 and 3, while the leaf on the left side connected with loop 4 and the proximal colon (Fig. 5a,b and 3D-PDF CS20).

#### The appearance of tertiary loops (week 8)

**Fig. 6:**
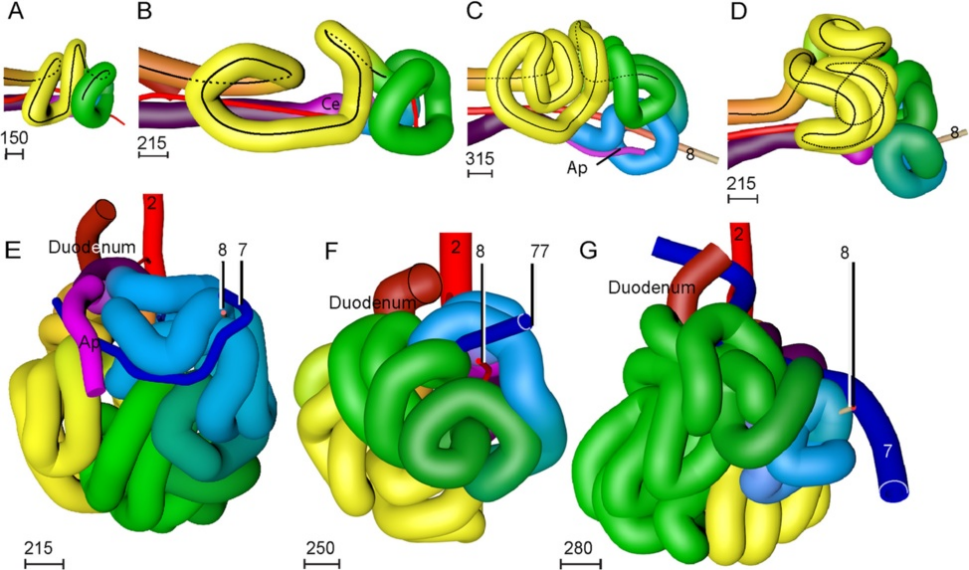
Tertiary loops arise from the 8^th^ week onwards and exhibit variation in number and position. Panels **a**-**d** show caudal views of the progressive folding of the apex of the 2^nd^ secondary loop (*coded yellow*) that results in tertiary loops. Panel **a**: CS20 (462); panel **b**: CS21 (4090); panel **c**: CS22 (H983); panel **d**: CS23 (s4141). The tip of the extending loops remains close to the mesenteric rod. Panels **e**-**g** show ventral views of three CS23 embryos of increasing size from left to right (s48, s4141, s9226). Note the variation in number and position of the tertiary loops and the fairly rapid movement of the 2^nd^ secondary loop from *right* (**e**) to *left* (**g**). For color codes, see Figure Legends. The scale bars (units in μm) show the diameter of the intestines with their surrounding mesenchyme in that panel. An interactive 3D-PDF is available online (3D-PDF CS23)

The length of the small intestine increased ~6-fold during CS20-23 (Fig. 6) with all secondary loops lengthening evenly. In addition, the radial length of the mesenteric leaves of the secondary loops increased, so that each secondary loop now clearly had its own leaf (3D-PDF CS23; compare Fig. 5b,c). The 3^rd^ and 4^th^ loops remained separated by the central axis, formed by the mesenteric rod, right vitelline vein, SMA, and periductal mesenchyme. The mesenteric leaves associated with the secondary loops remained recognizable during subsequent development and, thus, formed landmarks for these loops. The continued longitudinal growth was further accompanied by the appearance of tertiary loops within the secondary loops (Fig. 6a-d). The tertiary loops differed from the secondary loops in that their deposition varied between different embryos of similar stage (Fig. 6e-g). Of note, tertiary loops only developed within secondary loops with a mesentery. Accordingly, the proximal (future duodenal) part of the 1^st^ secondary loop (coded red) did not develop tertiary loops. Interestingly, the 2^nd^ secondary loop (coded yellow) assumed a left-sided position inside the hernia during the formation of tertiary loops (Fig. 6e-g). Although this change in position is obviously important for its future position in the abdomen (see: “Resolution of the hernia”), we have not identified any external cause for it.

#### The branching pattern of the superior mesenteric artery

**Fig. 7:**
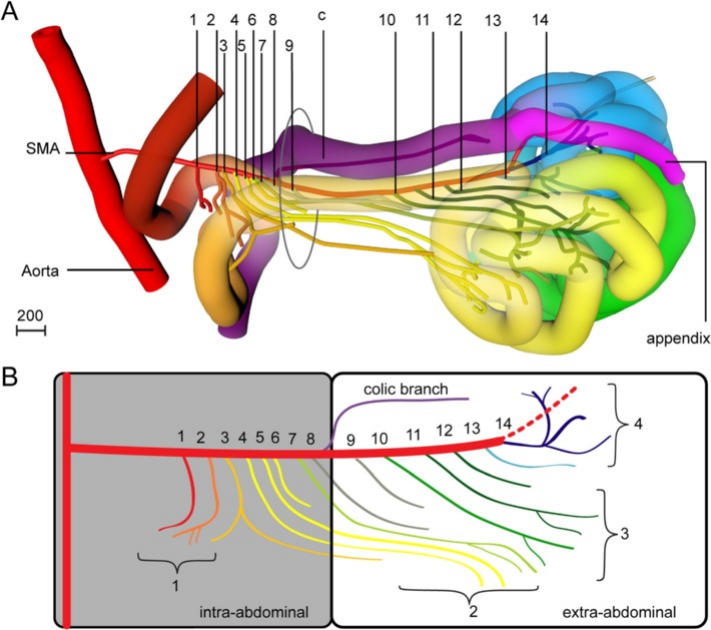
Branches of the superior mesenteric artery are stable landmarks for the midgut. *Right*-*sided* view of the reconstructed midgut and arterial tree of the SMA of a CS23 embryo (s48, panel **a**) and the corresponding schematic representation of the arterial tree (panel **b**). The most proximal 8 branches originated intra-abdominally and supplied the 1^st^ and 2^nd^ secondary loops. The third branch is typically bifurcated and perfused the intestine in the neck of the umbilical hernia. Distances between the 5 branches that originated extra-abdominally were longer. These branches supplied the 3^rd^ secondary loop. Distally, the SMA formed a vascular “broom” that supplied the 4^th^ secondary loop and cecum. Scale bar unit: μm. Interactive 3D-PDFs are available online (3D-PDF CS23, 9.0, and 9.5 WKS)

The SMA, which was reconstructed in five specimens from 8 to 10 weeks of development, showed a consistent branching pattern, with 10–13 small-intestinal branches originating from the caudal side of the artery and 1–3 smaller colonic branches from its cranial side (Fig. 7a,b). The 7–8 caudal branches with an intra-abdominal origin at CS23 supplied the 1^st^ and 2^nd^ secondary loops. The 3^rd^ caudal branch was of particular interest, because it supplied both intra- (distal part of the 1^st^ secondary loop; coded orange) and extra-abdominal (2^nd^ secondary loop; coded yellow) portions of the small intestine (Fig. 7a,b). The portion of the SMA passing the umbilical ring did not give off caudal branches, which probably explains the short gap between the intra- and extra-abdominal branches of the SMA. Distances between extra-abdominal branches were larger than those between intra-abdominal branches. Extra-abdominal branches 10–12 supplied the 3^rd^ secondary loop (coded green) and the terminal branches the 4^th^ secondary loop (coded blue). The periductal mesenchyme was still present as the landmark defining the apex of the midgut. A colic branch consistently arose on the cranial side of the SMA between the 7^th^ and 9^th^ caudal SMA branches. Because of the constant branching pattern of the SMA, these branches were suitable landmarks to follow the fate of the intestinal coils during and after the return of the herniated intestinal loops in the 9^th^ week of development.

### Resolution of the hernia (week 9)

**Fig. 8:**
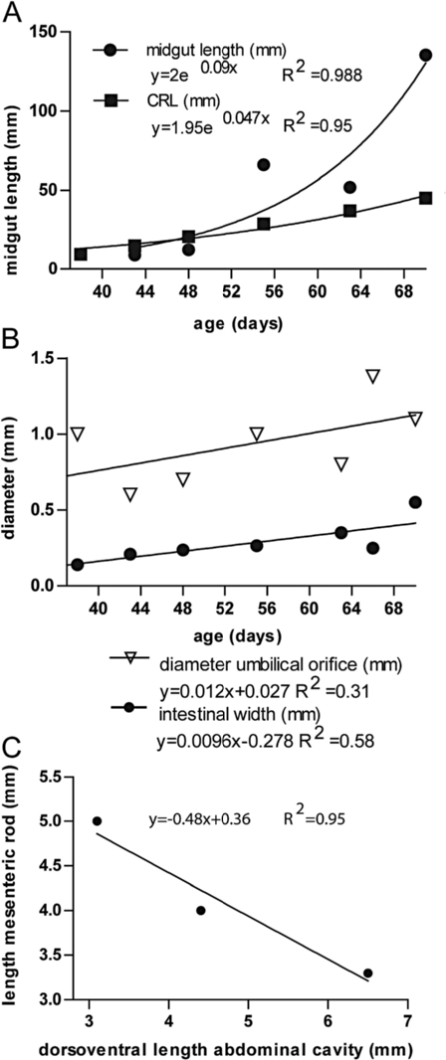
Changes in length and diameter of the small intestine and umbilical orifice. Panel **a**: The midgut (*circles*) increases much faster in length than the entire embryo (*squares*). Panel **b**: The diameter of the umbilical orifice (*open triangles*) is ~4-fold larger than the outer diameter of the gut tube (*circles*) throughout the herniation period. Panel **c**: length mesenteric rod vs dorsoventral depth of abdominal cavity

While the crown-rump length (CRL) of the embryo and the length of the small intestine increased ~4- and >10-fold, respectively, between 5 and 9 weeks of development (Fig. 8a), the diameters of the intestinal tube and the umbilical orifice both increased only ~1.5-fold (Fig. 8b). Since the diameter of the umbilical orifice is ~4-fold larger than the outer diameter of the gut tube throughout the period of study, ~8 gut loops would be able to pass at once if the mesentery is not included, indicating that the umbilical orifice remained large enough to allow passage of intestinal coils, even shortly after intestinal return. Intestinal return from the hernial sac could also be triggered by an increase in free abdominal space. However, the length of the intestine increased 1.5 fold more rapidly than the abdominal width. Furthermore, the liver continued to occupy a large portion of the abdominal volume, as its right inferior border continued to reach the level of the lumbar vertebrae L3–4. In addition, the ratio of the volume of the left and right liver lobes did not change between 5 and 10 weeks development. Finally, as Fig. 8c shows, we observed that the dorso-ventral “depth” of the abdomen (measured as the distance between the aorta and the umbilical orifice) increased ~2-fold between 8.0 and 9.5 weeks of development, while the mesenteric rod became shorter in the same period. This differential increase in the depth of the abdomen and the length of mesenteric rod should facilitate and may even initiate intestinal return from the hernial sac.

#### The post-herniation position of the secondary loops

**Fig. 9:**
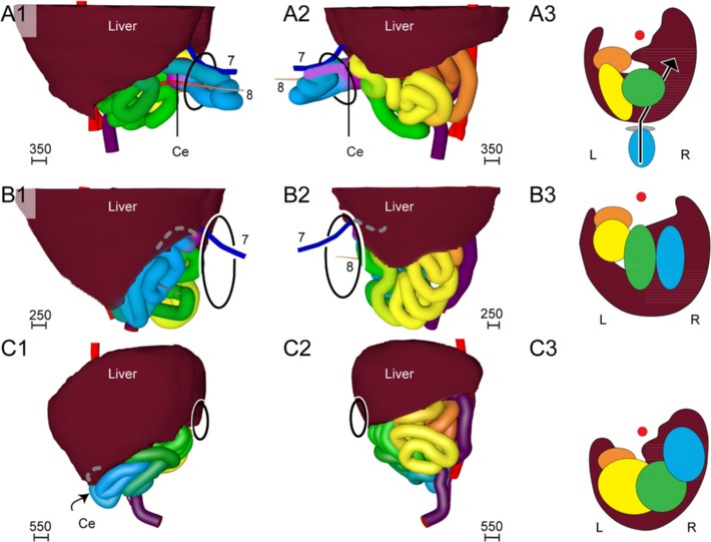
The ileum assumes its right-sided position only after the umbilical herniation is completely resolved at 10 weeks development. *Right*-*sided* (panels **a1**-**c1**), left-sided (panels **a2**-**c2**) views and caudal views (panels **a3**-**c3**) of schematic representations of reconstructions of 9.0 week (s89; *top row*), 9.5 week (s57; *middle row*), and 10 week (s1507; *bottom row*) embryos. The ovals indicate the umbilical orifice. The loops of the 4^th^ secondary loop (blue) and the appendix are still inside the hernial sac at 9 weeks, occupy an intra-abdominal position close to the hernial rim at 9.5 weeks, and have attained their definitive positions (cecum and appendix at L4) at 10 weeks. The appendix is hidden behind the liver in panels *b* and *c*, and is indicated with a gray dashed line. L: left; R: *right*. The scale bars (units in μm) show the diameter of the intestines with their surrounding mesenchyme in that panel. Interactive 3D-PDFs are available online (3D-PDF 9.0WKS and 9.5 WKS)

**Fig. 10:**
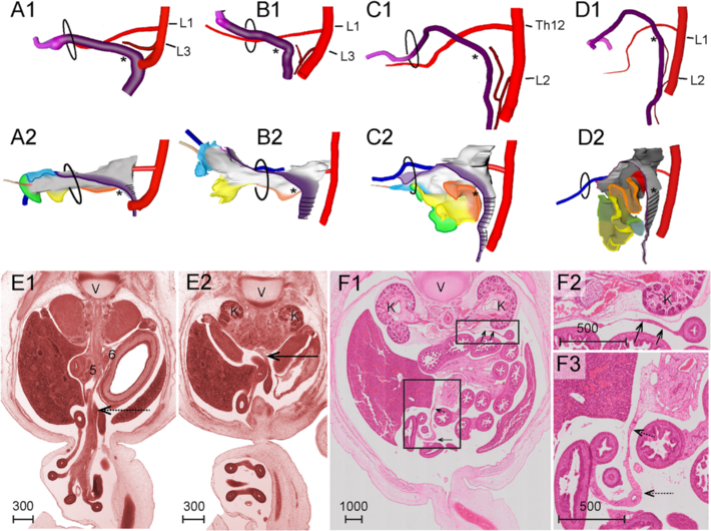
Changes in colonic topography during the herniation and postherniation period. *Left*-*sided* views of reconstructions of the colon (panels **a1**-**d1**) and the colonic mesentery (panels **a2**-**d2**; *purple edge*) of CS18 (panel **a**; s97), CS23 (panel **b**; s48), 9.0 weeks (panel **c**; s89), and 9.5 weeks (panel **d**; s57) embryos. Note the boundary (*) between the *horizontal*, herniating proximal colon and the *vertical*, intra-abdominal distal colon at the colic bend. The asterisk also identifies the boundary between the cranial branches of the SMA (originating at Th12/L1) and the ascending branch of the inferior mesenteric artery (originating at L2/L4). The appendix and a few distal coils were still located in the hernial sac at 9.0 weeks and had just passed the hernial rim at 9.5 weeks. Panels **e** and **f** show the mesentery of the proximal colon of a CS18 (s97) and a 9.5 week embryo (s57), respectively. The mesentery of the proximal colon is attached to the mesenteric rod (stippled *arrows* in **e1** and **f1**,**3**), whereas that of the distal colon is attached to the dorsal midline (*arrows* in **e2**, **f1**,**2**). Note *leftward* change in position of distal colon and mesentery between panels **e2** and **f**. Interactive 3D-PDFs are available online (3D-PDF CS14, CS16, CS20, CS23, and 9.0 and 9.5 WKS)

**Fig. 11:**
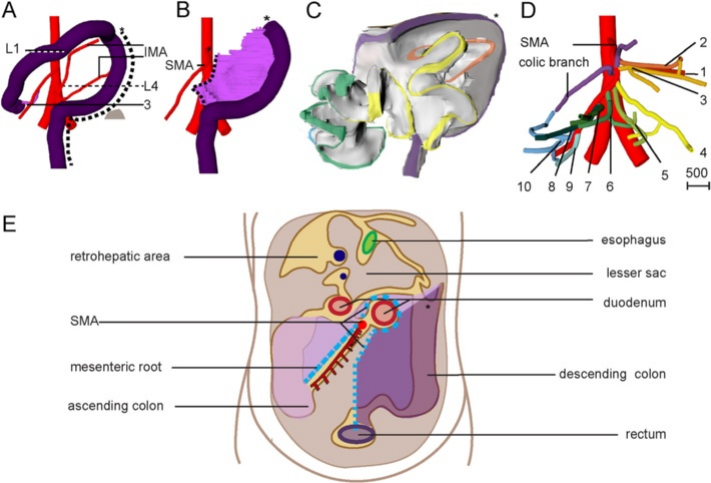
Similarity of the colon and its mesentery and vessels in the 10 week embryo and the adult. Ventral views showing the entire colon (panel **a**), the distal colon with its mesentery (panel **b**), the entire mesocolon (panel **c**), and the winding staircase appearance of the branches of the superior mesenteric artery (panel **d**) of a 10.0 week (s1507) embryo. Note presence of the colic bend (*; future splenic flexure) and absence of hepatic bend. Scale bar unit: μm. Panel **e** shows, for comparison, the position of the colonic mesentery in the adult, with the parts of the mesocolon of the ascending and descending colon that have merged with the posterior body wall shown in light and dark purple, respectively

The 1^st^ secondary loop increased substantially in length and continued to expand in a leftward direction caudally of the mesenteric rod. The 2^nd^ and 3^rd^ secondary loops moved back into the abdominal cavity between 8.5 and 9.0 weeks of development to occupy left-sided and mostly median positions, respectively (Fig. 9a; 3D-PDF 9.0 WKS). The colon co-migrated with these loops as demonstrated by the finding that the cecum had moved inward before the 4^th^ secondary loop of the small intestine and the appendix (Figs. 9a and 10c). The 4^th^ secondary loop and cecum returned to a ventral midline position in the abdominal cavity at 9.5 weeks (Fig. 9b). Between 9.5 and 10.0 weeks, the 4^th^ secondary loop, cecum and appendix moved rightward and only slightly caudal to their definitive positions near the iliac crest (corresponding to lumbar vertebra L4; Figs. 9c and 11a; 3D-PDF 9.5 WKS). After the complete return of the herniated intestine, the empty hernial sac persisted for at least a week, indicating that the newly attained intra-abdominal position of the intestines was not metastable. The described deposition of the small intestine and its mesentery from the upper left to the lower right part of the abdominal cavity was reflected in the winding staircase appearance of the approximately 10 (caudal) branches of the SMA (Fig. 11d) and represents its definitive position.

### Development of the colon

The colon did not develop secondary loops. In agreement, the relative contribution of the colon to the total intestinal length declined with age (from ~50 % of small intestinal length at CS14 to ~15 % at 10 weeks). The cecum was located close to the dorsal body wall at CS14 (Fig. 1a,b), herniated at CS16 (Fig. 3e), and occupied a left-sided position during the entire herniation phase, but remained close to the midline (Fig. 4). The mesentery of the proximal colon connected to the mesenteric rod (Fig. 10e1, f1 and f3), whereas that of the distal colon attached in the midline to the dorsal body wall from the kidneys (Th12) downward (Fig. 10e2, f1 and f2). The transition of the proximal colon into the distal colon was located where the mesocolon passed as a “roof” over the vitelline vein, SMA, and duodenojejunal junction (Fig. 10a-d). It was marked by the boundary of the vascular territories of the superior and inferior mesenteric arteries, which were identifiable from CS15 onwards. After CS23, when the branches of the IMA become unambiguously identifiable the left colic bend can be clearly distinguished. The descending colon and its mesentery moved leftward between 8 and 10 weeks of development, concomitant with the leftward increase in length of the 1^st^ secondary loop (Fig. 4). The proximal colon and its mesentery followed the course of the central mesenteric rod and the SMA from a still medial position and dorsoventral orientation at 9.5 weeks (Fig. 10d) to a diagonal and coronal position at 10.0 weeks (Fig. 11a-c). In the 10^th^ week, the proximal colon did not have separate ascending and transverse portions yet, and accordingly a hepatic flexure was not yet identifiable. Figure 11e shows, for comparison, the position of the colonic mesentery in the adult, with the parts of the mesocolon of the ascending and descending colon that still have to merge with the posterior body wall shown in light and dark purple, respectively.

## Discussion

We studied the midgut during its looping and herniation phases of development and distinguished 3 generations of midgut loops. The topography of the primary and secondary loops was constant, but that of tertiary loops not. The primary loop changed from a sagittal to a transverse orientation due to the descent of ventral structures over at least 8 segments when the body axis was still helical. The 4 secondary loops determined the definitive topography of the intestine. The 1^st^ secondary loop (duodenum and proximal jejunum) assumed its left-sided position during herniation. The 2^nd^ secondary loop (distal jejunum) assumed a left-sided position inside the hernia just prior to return, while the 3^rd^ and 4^th^ secondary loops retained near-midline positions. Intestinal return into the abdomen resembled a backward sliding movement. Only after its return into the abdominal cavity, the 4^th^ secondary loop (distal ileum and cecum) descended to the right lower abdomen. Our description shows that the “en-bloc rotation” model of midgut development is a fallacy of schematic drawings.

### The midgut as a structural entity

The midgut is usually defined as the portion of the gut that is perfused by the SMA and that shows the characteristic features of herniation and rotation [8]. Earlier definitions described the midgut as the intestinal loop between the duodenojejunal flexure cranially and the colic bend (or angle or flexure) caudally [6, 16]. These definitions leave space for arguments about the proximal and distal boundaries. If the boundary of the perfusion areas of the celiac trunk and SMA is taken as criterion for the junction of the foregut and midgut, the proximal boundary is positioned in the perfusion area of the pancreaticoduodenal arteries [17, 18]. Furthermore, the ventral mesentery and the glands that penetrate the muscularis muscle (liver, dorsal and ventral pancreas, Brunner’s glands) end where the duct of the common bile duct and ventral pancreas enter the duodenum. Vascular supply, caudal end of the ventral mesentery, and intestinal architecture appear strong arguments to locate the junction between the caudal foregut and midgut at the ventral pancreatic duct, that is, midway along the descending part of the definitive duodenum. If the remainder of the duodenum represents, by consequence, the most cranial portion of the midgut, it never acquires the thin dorsal mesentery that develops more caudally from CS14 onwards. We hypothesize that a relatively thin dorsal mesentery identifies parts of the gut with rapid longitudinal growth. Interestingly, we did not observe tertiary looping in the distal duodenum, suggesting that tertiary looping reflects rapid longitudinal growth and requires the presence of a thin mesentery. The (left) colic bend as caudal boundary of the midgut is present as the gradual transition of the horizontal into the vertical portion of the colon during herniation, but can be unambiguously identified after the main branches of the superior and inferior mesenteric arteries become identifiable at CS23 and even more so after the bend has become acute after week 13.

### The developmental origin of the convoluted small intestine

Frazer and Robbins stated that the midgut developed between fixed proximal and distal boundaries: the duodenojejunal junction was fixed by the ligament of Treitz (LOT) and the colic angle by a “retention band” [6]. Snyder and Chaffin considered both bends as growth zones instead [19]. We and others observed a rapid longitudinal growth of the entire small-intestinal part of the midgut and the coincident thinning of the mesentery [20]. The midgut formed 3 generations of loops, each with their own characteristics.

#### Primary loop

The primary loop encompasses the entire midgut. Its asymmetric growth has been ascribed to laterality in gene expression [21], and asymmetric outgrowth of the mesentery due to extracellular-matrix and cytoskeletal remodeling [22–25]. The correlation between heterotaxia syndromes and malrotation also suggests a genetic component in intestinal looping [26]. However, the chirality of heart looping in chicks was recently shown to depend on the turning of the head [27], that is, upon a biophysical factor [28]. When the primary loop was still a midline structure at CS14, its limbs followed the helical shape of the body axis. Concomitant with the descent of the ventral thoracic and upper abdominal structures after CS13 (also reported by [10, 29–31], more cranial midline structures acquired a right-sided position and more caudal structures a left-sided position relative to the midline. These findings imply that the change in the position of the primary loop, like the heart loop, depends on the chiral growth of the body. We found no evidence for an effect of an asymmetric expansion of the liver and associated positional changes of the vasculature [16, 32], because the liver already has an asymmetric shape prior to the rightward shift of the proximal limb of the primary loop [33].

#### Secondary loops

The small intestinal portion of the primary loop was further divided into secondary loops according to a strict spatiotemporal pattern during the late 6^th^ and early 7^th^ week. This second generation of intestinal loops and their very constant distribution were already described two centuries ago [10–12], but have since disappeared from descriptions, probably because their presence did not accommodate the prevailing theories of intestinal malrotation (see: “Implications for malformations”). The secondary loops probably develop, like the primary loop, as a result of the rapid growth of the intestinal tube, but the exact number (four) and predictable growth pattern of the secondary loops suggests a genetic regulation.

#### Tertiary loops

The secondary loops formed additional, tertiary loops after the second half of the 8^th^ week of development. Tertiary loops did not have a predictable constant distribution pattern and their formation coincided with an acceleration of the longitudinal growth of the small intestine. In a recently proposed model of gut development, regular loops emerged when the relative growth rates and elasticity of the gut tube and its mesentery differed [14]. Even though both the length of the gut and that of the mesentery increased, the individual domains of the four secondary loops remained identifiable since the mesenteric leaves were shorter in the transition areas between these domains. Mall already showed that the 4 domains of the secondary loops could still be identified in ~50 % of adult cadavers [10]. We could confirm his findings in a smaller number of cadavers. If not all domains could be identified, there were fewer or one was of smaller size.

### What triggers intestinal return?

Prior to return into the abdominal cavity, the longitudinal growth of the intestine was much more rapid than that of the embryo, but its diameter increased at the same rate as that of the hernial connection with the abdominal cavity and allowed the passage of several intestinal loops at once. We and others showed in rare intermediate stage embryos (9.5 weeks) that the intestine returned in a proximodistal fashion, with the distal jejunal loop (yellow) first [34], then the proximal ileal loop (green) and cecum, and the distal ileal loop (blue) and appendix last (Fig. 9a). The deep incisures between the mesenteries of the secondary loops further suggested that the secondary loops could move independently of each other. This architecture could facilitate phased return of the loops through the umbilical orifice. These size considerations imply that, even if tertiary loops can only partially uncoil, the width of the hernia neck does not appear to determine the time window for intestinal return, as is often hypothesized [7]. Although we are not aware of experiments to explore the return mechanism, we hypothesize that the decrease of the length of the mesenteric rod relative to the dorso-ventral depth of the abdomen is responsible for the proximodistal sequence in the return of the intestinal segments to the abdominal cavity. This mechanism was first proposed by Pernkopf in 1925 [11, 12].

### Does the midgut rotate?

**Fig. 12:**
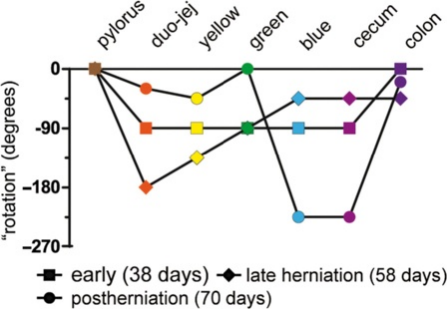
“Rotation” of the midgut relative to the superior mesenteric artery. The diagram shows the changes in position (“rotation”) of the indicated intestinal structures relative to the SMA as seen from ventral. Cranial relative to the SMA represents 0° and the gut “rotates” counterclockwise. The squares represent rotation associated with the primary loop. The diamonds show the degree of rotation between 5.5 and 8.5 weeks, while the circles show the degree of rotation during and immediately after intestinal return (9^th^ week). Only rotation and not distance to the SMA are shown, so that rotation during intestinal return appears extensive, whereas the change in position is only minor. We conclude that the intestines do not “rotate” but “slide” from the umbilical orifice to the lower-right abdominal cavity

**Fig. 13:**
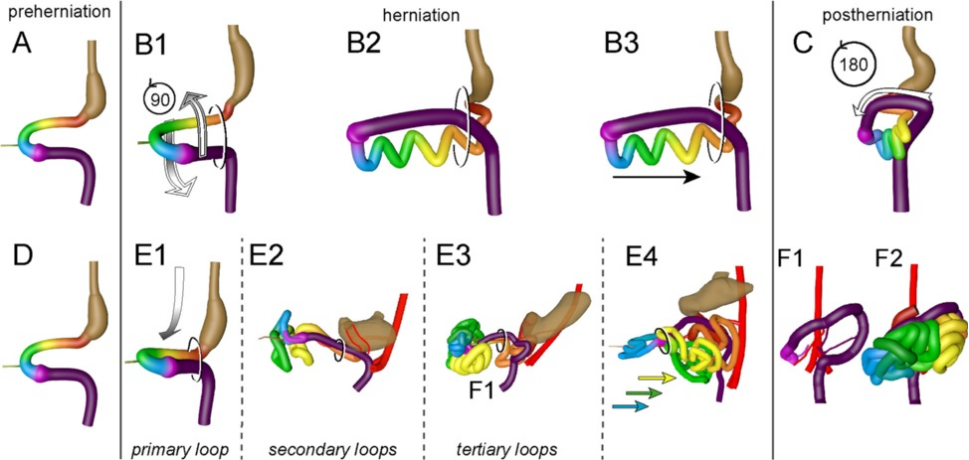
Graphical summary of the “en-bloc rotation” and our hierarchical model of gut morphogenesis. Panels **a**-**c** show the classic “en-bloc rotation” model of gut morphogenesis. From panel **a** to **b** (*left*-*sided views*) the midgut loop rotates 90° in a counterclockwise direction, so that its position changes from midsagittal (**a**) to transverse (**b1**). The small intestine forms loops (**b2**) and slides back into the abdomen (**b3**) during resolution of the hernia. Meanwhile, the cecum moves from the *left to the right side*, which represents the additional 180° counterclockwise rotation of the intestine (**c**, *ventral view*). The present study shows that the gut acquires its definitive shape by the hierarchical development of primary, secondary, and tertiary loops (panels **d**-**f**). The descent of the proximal midgut in the still helically shaped body rather than rotation accounts for the change in position of the primary loop from midsagittal (**d**, *left*-*sided view*) to transverse (**e1** *left*-*sided view*). During the herniation phase, 4 secondary loops develop in a strict spatiotemporal fashion in the small intestine (**e2** *left*-*sided view*). Tertiary loops develop within the secondary loops (**e3**) and these domains slide in a proximodistal fashion back into the abdomen, with the distal ileum and appendix last (**e4** *left*-*sided view*). Just after return, the cecum is found medially, just dorsal to the umbilical opening. Within 4 days the cecum then assumes its *right*-*sided*, more caudal and dorsal position (**f1** (colon only), **f2** (colon and small intestine); *ventral views*)

We have, of course, asked ourselves the question whether the change in position of the duodenojejunal junction and the cecum relative to the axis of the SMA represented a 270° rotation. Rotation is usually described to occur in 2 phases, that is, during formation of the primary loop and upon intestinal return into the abdominal cavity [6, 16], but sometimes an intermediate stage that represents the growth of the secondary loops is included [8]. We also mapped the positions of the 4 secondary loops relative to the SMA as degrees of rotation when they first develop (5.5 weeks), during intrahernial growth (between 5.5 and 8 weeks), and after return (>9.0 weeks). Figure 12 shows that the rotation of the primary loop is experienced by all parts of the midgut. Although the outcome of our model does not differ from the “en-bloc” rotation model in this phase of development, it proposes for the first time a potential mechanism for the initiation of asymmetric gut development, namely the descent of the distal foregut and its derivatives in embryos with a still helical body axis. The subsequent topographical changes of the intestine relative to the SMA during late herniation are most pronounced in the proximal duodeno-jejunal loop (orange) which extends caudal to the SMA in a leftward direction over a 3-week period, suggesting it represents a period of local growth. Thereafter, the position of this segment hardly changes in position. In contrast, the distal ileal loop (blue) hardly changes in position relative to the SMA during late herniation, but does so within a few days after return in the abdominal cavity. As Fig. 9 shows, this latter change in position is due to a movement of this part of the gut from midsagittal and ventral to right-lateral and more dorsal. Even though the rotational change of this part of the intestine after intestinal return appears extensive, the linear change in position is only minor. If one insists on using the term rotation for this movement, it would be largely around a craniocaudal axis (in the transverse plane) rather than a dorsoventral axis (frontal plane). In view of the brief time window and orientation of the apparent rotational axis, we conclude that the distal ileum and cecum “slide” rather than “rotate” as from the umbilical orifice to the lower-right abdominal cavity. “En-bloc rotation” is a conceptually simple and, therefore, attractive model of intestinal development, but the very different degrees, rates, and developmental timing of the apparent rotation of different parts of the midgut shows that this model cannot be upheld. Our present study shows that hierarchical looping is a viable new model to describe key morphogenetic events in intestinal development (for a comparison of both models, see Fig. 13).

### Implications for malformations

By far the best-known malformation of the intestine is malrotation. If problems with intestinal rotation are taken as the underlying mechanism, reversed rotation and complete nonrotation are the extreme conditions of the spectrum of malrotations [35]. Reversed rotation is a rare anomaly in which colon and duodenum rotate clockwise instead of counterclockwise in relation to the SMA, so that the transverse colon ends up posterior to the superior mesenteric vessels [36]. We hypothesize that the early helical growth of the embryo is reversed in these cases. If nonrotation is diagnosed, the cecum and ascending colon have a left-sided instead of the normal right-sided position, while the position of the ligament of Treitz (LOT) is unaffected. This intestinal configuration resembles the configuration before the “slide” of ileum and proximal colon from the umbilical orifice to the lower-right abdominal cavity. The remaining malrotations are classified as either typical or atypical [4]. A typical malrotation is defined by a right-sided position of the LOT, whereas an atypical malrotation has a left-sided LOT relative to the vertebral column [3, 35]. The position of the cecum is subhepatic in the vast majority (~85 %) of both typical and atypical malrotations [35, 37], that is, the ascending colon is too short. The position of the LOT can be regarded as the marker for the development of the 1^st^ secondary loop and the position of the cecum as marker for the 4^th^ secondary loop [19]. Abnormal positions of the LOT and cecum are not necessarily linked [3, 19].

Based on our study, we hypothesize that growth restriction of secondary loops underlies the development of malrotations. A right-sided position of the LOT implies that the growth of the 1^st^ secondary loop is impaired. Similarly, we hypothesize that a cranial position of the cecum and short ascending colon imply impaired development of the 4^th^ secondary loop and proximal colon. The cecal position in malrotations resembles that at 9.5 weeks of development, so just after intestinal return, when it occupies a subhepatic position and the proximal colon is still relatively short. It is not surprising that underdevelopment of the 1^st^ and 4^th^ secondary loops produces a more pronounced phenotype than of the 2^nd^ or 3^rd^ secondary loops, because underdevelopment of the latter loops would “only” present as short bowel syndrome. In agreement, short bowel syndrome occurs in combination with intestinal malrotation as a genetic disease [38–40]. Variation in the degree of development of the respective secondary loops would also explain the variance in the size of the domains of the small intestine in adults [10].

What could cause growth retardation of the intestine? Although we can only speculate about the cause(s), vascular incidents are an appealing option. A rare but instructive congenital malformation is the “apple peel” small bowel. This malformation is characterized by jejunal or ileal atresia and absence of the dorsal mesentery [41, 42]. In some cases of jejunal or ileal atresia, squamous epithelial cells, lanugo hair, or bile droplets were reportedly found distal to the atresia and even between atretic segments [43, 44], suggesting the atresia developed after bile production started. These congenital malformations were plausibly attributed to intestinal injuries after e.g., vascular accidents. We hypothesize that similar accidents can cause insufficient growth of the secondary loops during herniation or shortly after return. This hypothesis may also explain the presence of Ladd’s bands: after the injury, tissue repair entails the development of scar tissue.

## Conclusions

Intestinal morphogenesis is characterized by 3 phases of looping, each with a distinct underlying mechanism. The secondary loops of the small intestine develop according to a highly predictable pattern. We hypothesize, based on published accounts of malrotations, that the pathology associated with these malformations results from incomplete development of the secondary loops.

## Methods

### Specimens

This study was undertaken in accordance with the Dutch regulation for the proper use of human tissue for medical research purposes. We included anonymized specimens from the historical collections of embryos and fetuses of the Departments of Anatomy and Embryology, Leiden University Medical Center, Leiden, and the Academic Medical Center, Amsterdam, The Netherlands, and the Carnegie Collection, Washington D.C., USA (obtained via the Digitally Reproduced Embryonic Morphology (DREM) project) that were donated for scientific research. Only specimens with an intact umbilicus were selected for reconstruction. The criteria of O’Rahilly [15] were used to determine the Carnegie stage of development. In total, 19 embryos representing a gradual progression of intestinal development were reconstructed (Table 1). In addition, we studied other serially sectioned embryos of the LUMC and AMC collections that were not reconstructed. Finally, we compared our reconstructions with the reconstructions in the historic reports of Mall [10], Frazer & Robins [6], and Pernkopf [11, 12, 31]. Brain development [45], and the return of the physiological hernia between 9.0 and 9.5 weeks of development [46, 47] were used to estimate the age of the embryos after Carnegie stage 23. Specimens of 8–10 weeks development will also be referred to as embryos.

**Table 1:** Reconstructed embryos

Carnegie stage/age	Embryo identification	Embryo collection	Plane of sectioning	Stain	Voxel size (μm)
Early 14	s2201	AMC	Transverse	HA	1*1*3
Late 14	s5029	AMC	Sagittal	HA	1.1*1.2*30
Late 14	6502	Carnegie	Transverse	Souza	1.06*1.06*100
15	2213	AMC	Transverse	HA	3.25*3.25*20
16	s5032	AMC	Sagittal	HA	6.5*6.5*13
Late 16	6517	Carnegie	Transverse	Carmine	5.3*5.3*32
17	6520	Carnegie	Transverse	Carmine	2.6*2.6*60
18 early	s97	AMC	Transverse	HE/Azan	3.25*3.25*30
18 late	4430	Carnegie	Transverse	Carmine	6.6*6.6*80
20	462	Carnegie	Transverse	Carmine	3.33*3.33*80
20	s2025	LUMC	Sagittal	HE	4.0*4.0*21
21	4090	Carnegie	Transverse	Carmine	11*11*80
22	H983	Carnegie	Transverse	HE/trichrome/silver	27*27*50
Early 23	s48	LUMC	Transverse	HE	5.2*5.2*30
Mid 23	s4141	AMC	Transverse	HA	6.5*6.5*10
Late 23	9226	Carnegie	Transverse	Azan	12.3*12.3*120
9.0 weeks	s89	LUMC	Transverse	HE/Azan	6.5*6.5*30
9.5 weeks	s57	LUMC	Transverse	HE	5.2*5.2*20
10.0 weeks	s1507	AMC	Transverse	HA	6.5*6.5*197

### Image acquisition and processing

The embryos that were reconstructed are shown in Table 1. Digitized images of serial sections of DREM embryos are directly available via the Virtual Human Embryo project [48, 49]. Serial sections of the embryos from the AMC and LUMC collections were digitized at high resolution with an Olympus BX51 scanning microscope and dotSlide software (Olympus, Zoeterwoude, the Netherlands). Resized JPEG images were converted to grey-scale images with Photoshop CS5. The image resizing factor was kept minimal to preserve detail and correlated with embryo size (Table 1).

### 3D reconstruction

The 3D reconstructions were generated with Amira™ (version 5.5; base package; FEI Visualization Sciences Group Europe, Mérignac Cédex, France). Serial sections were first aligned automatically with the least-squares method and then manually adjusted to account for curvature and rotation of the body axis with the help of photographic, MRI and ultrasound images of age-matched embryos [50]; Aligned slices were resampled into the Amira mesh-file format. The intestine, mesentery, vessels and reference structures were segmented manually based on histologically identifiable contours. The outer muscular layer was used to delineate the esophagus and stomach, and the serosal layer of the intestine was used to delineate the intestinal tract to the transition of the hindgut from an intra- to a retroperitoneal position. Somites, intersegmental arteries, spinal ganglia, and vertebral bodies were used as landmarks for segmental levels.

To describe the precise topographic position of structures, landmarks such as somites, vertebrae, spinal ganglia, and intersegmental arteries were identified. The exact somite level was deduced from the position of the 7^th^ intersegmental artery (future subclavian artery) located between somites 10 and 11 [51–53]. Once vertebral development begins, the 7^th^ intersegmental artery is found in the loose zone of somite 11 and no longer between somites. As such, segments 1–7 correspond to C1-7, segments 8–19 to Th1-12, and segments 20–24 to L1-5.

### Reconstruction refinement

Polygon meshes were created from the segmented labels and exported to Cinema 4D (MAXON Computer GmbH, Friedrichsdorf, Germany). The meshes for the mesentery were optimized using deformers and sculpting, whereas the intestine and vessels were modeled with Bezier curves. All structures were modeled proportionally and scaled as indicated. Intestinal length was measured in Cinema4D by assessment of the spline length of the Bezier curves. The Cinema 4D files formed the basis for three-dimensional interactive PDFs [54].

### Supporting data

The data set(s) supporting the results of this article is (are) included within the article (and its Additional file 1).

## Additional file

Additional file 1:
**Supplemental Figures.** 3D PDF CS14, CS16, CS20, CS23, and 9.0 and 9.5 WKS. Interactive 3D PDFs are provided for CS14, CS16, CS20, CS23, and 9.0 and 9.5 WKS. See the supplement “3D PDF operation manual” for instructions how to use the interactive PDF tools. The color codes correspond to those used in the Figures (Legend Table). (PDF 394 kb)

## Abbreviations

CS: Carnegie Stage
CRL: Crown Rump Length
IMA: Inferior mesenteric artery
L: Lumbar
SMA: Superior mesenteric artery
Th: Thoracic
A: Allantois
Ap: Appendix
Ce: Cecum
D: Dorsal
Dp: Dorsal pancreas
GB: Gall bladder
He: Head
H: Heart
K: Kidney
L: Left
Li: Liver
NT: Neural tube
PG: Postanal gut
R: Right
SMA: Superior mesenteric artery
St: Stomach
T: Tail
V: Vertebra
V: Ventral
Vp: Ventral pancreas
Wd: Wolffian duct
