# Supplementary material for: The growth pattern of the human intestine and its mesentery
Source: BMC Dev Biol. 2015 Aug 22;15:31. doi: 10.1186/s12861-015-0081-x (PMC4546136; doi:10.1186/s12861-015-0081-x)

Maastricht University

The growth pattern of the human midgut and its mesentery

Jelly HM Soffers[1], Jill PJM Hikspoors[1], Hayelom Mekonen[1], S. Eleonore Köhler[1], Wouter H Lamers [1,2]

1: Department of Anatomy & Embryology, Maastricht University, NL 2: Tytgat Institute for Liver and Intestinal Research, Academic Medical Center, University of Amsterdam, NL

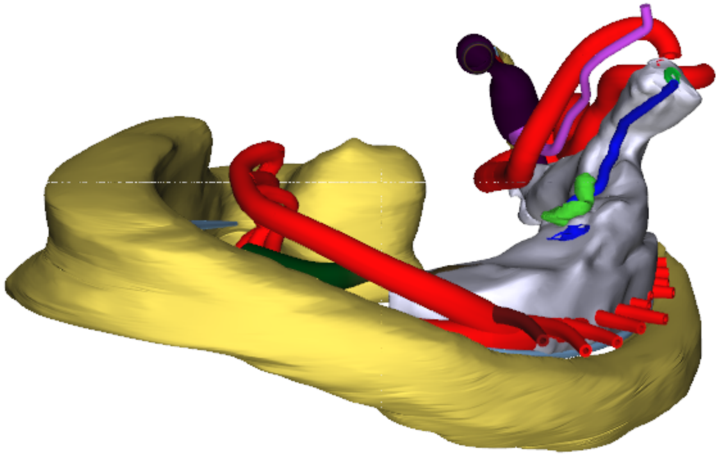

Maastricht University

The growth pattern of the human midgut and its mesentery

Jelly HM Soffers[1], Jill PJM Hikspoors[1], Hayelom Mekonen[1], S. Eleonore Köhler[1], Wouter H Lamers [1,2]
1: Department of Anatomy & Embryology, Maastricht University, NL 2: Tytgat Institute for Liver and Intestinal Research, Academic Medical Center, University of Amsterdam, NL

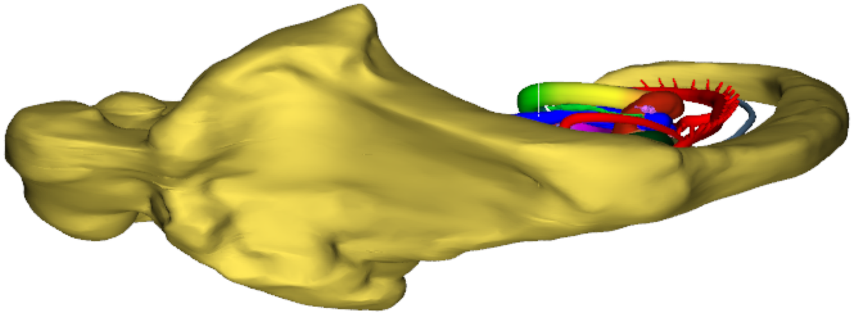

The growth pattern of the human midgut and its mesentery

Jelly HM Soffers[1], Jill PJM Hikspoors[1], Hayelom Mekonen[1], S. Eleonore Köhler[1], Wouter H Lamers [1,2]

1: Department of Anatomy & Embryology, Maastricht University, NL 2: Tytgat Institute for Liver and Intestinal Research, Academic Medical Center, University of Amsterdam, NL

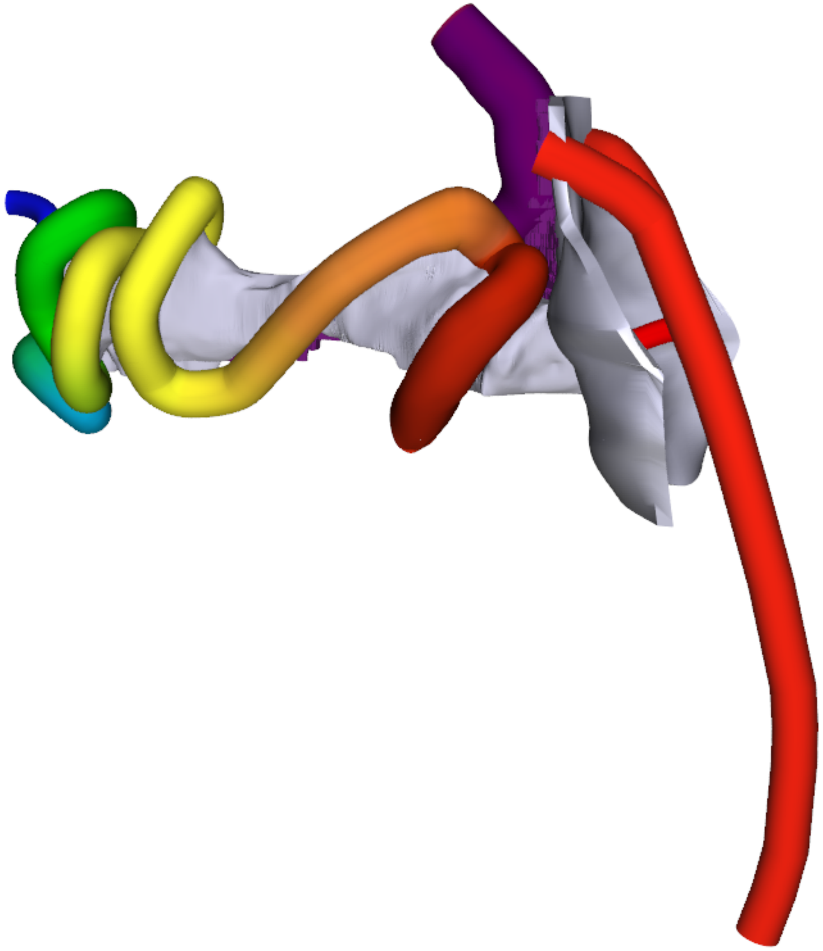

The growth pattern of the human midgut and its mesentery

Jelly HM Soffers[1], Jill PJM Hikspoors[1], Hayelom Mekonen[1], S. Eleonore Köhler[1], Wouter H Lamers [1,2]

1:Department of Anatomy & Embryology, Maastricht University, NL 2: Tytgat Institute for Liver and Intestinal Research, Academic Medical Center, University of Amsterdam, NL

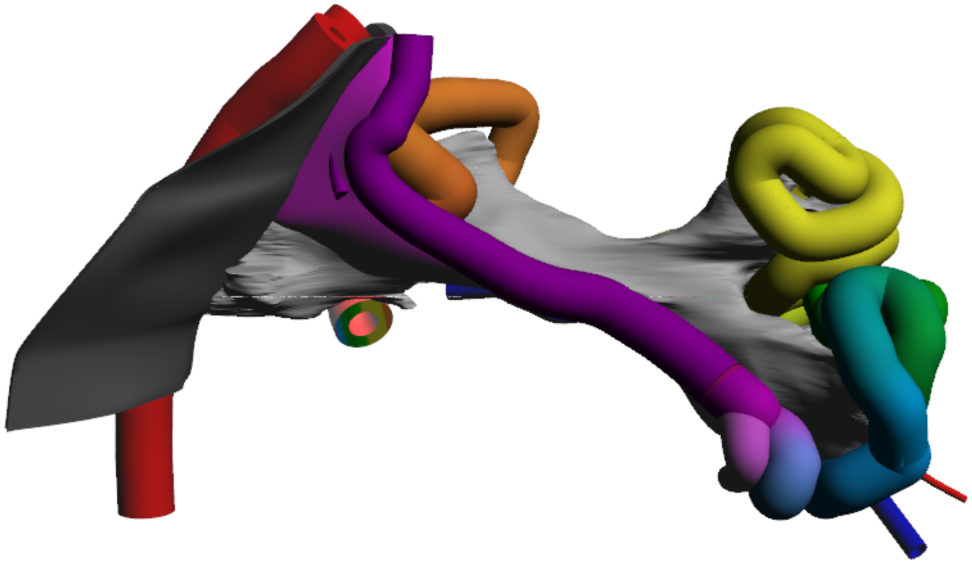

Maastricht University

The growth pattern of the human midgut and its mesentery

Jelly HM Soffers[1], Jill PJM Hikspoors[1], Hayelom Mekonen[1], S. Eleonore Köhler[1], Wouter H Lamers [1,2]

1: Department of Anatomy & Embryology, Maastricht University, NL 2: Tytgat Institute for Liver and Intestinal Research, Academic Medical Center, University of Amsterdam, NL

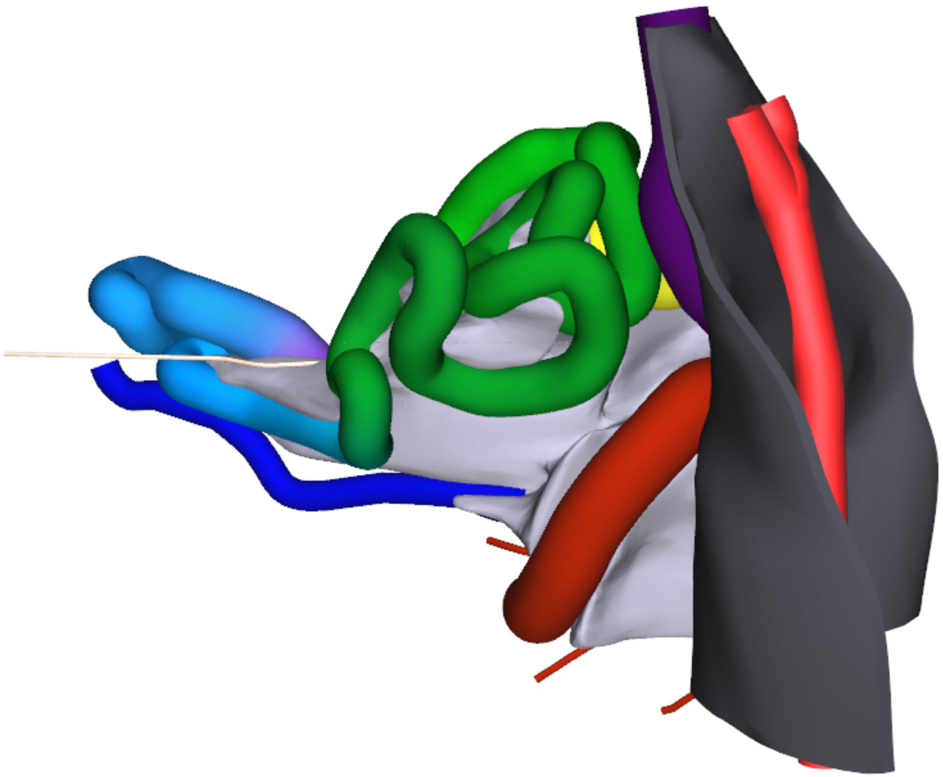

The growth pattern of the human midgut and its mesentery

Jelly HM Soffers[1], Jill PJM Hikspoors[1], Hayelom Mekonen[1], S. Eleonore Köhler[1], Wouter H Lamers [1,2]

1: Department of Anatomy & Embryology, Maastricht University, NL 2: Tytgat Institute for Liver and Intestinal Research, Academic Medical Center, University of Amsterdam, NL

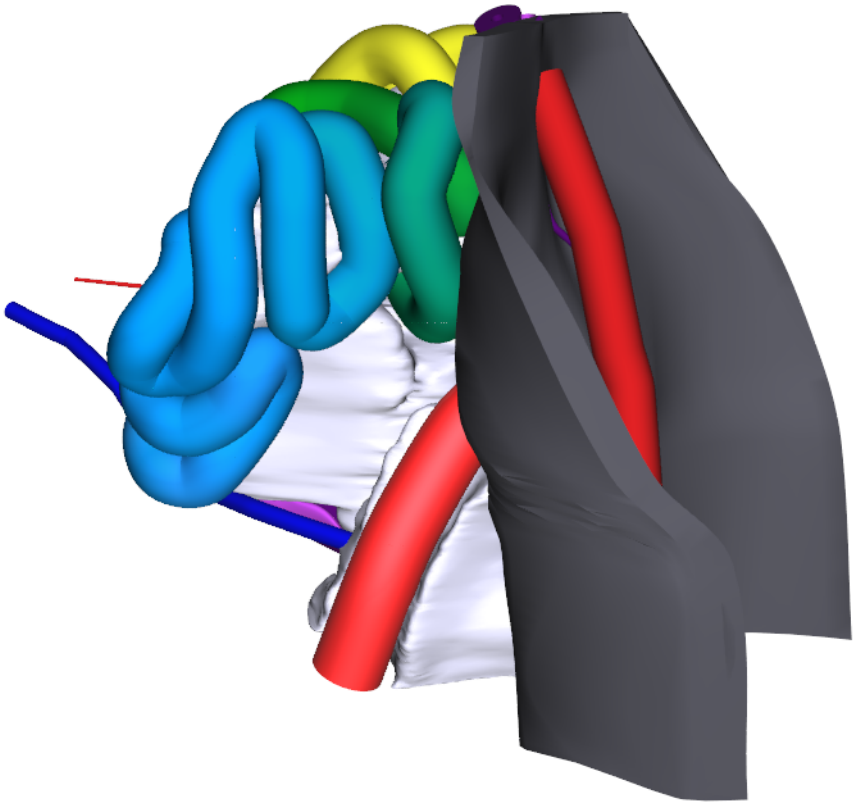

Supplement: Additional file 1: — Supplemental Figures. 3D PDF CS14, CS16, CS20, CS23, and 9.0 and 9.5 WKS. Interactive 3D PDFs are provided for CS14, CS16, CS20, CS23, and 9.0 and 9.5 WKS. See the supplement “3D PDF operation manual” for instructions how to use the interactive PDF tools. The color codes correspond to those used in the Figures (Legend Table). (PDF 394 kb) [file 12861_2015_81_MOESM1_ESM.pdf]
